# Baseline Severity of Sacroiliitis and Extensive Fat Metaplasia Predicts the Progression of Backfill at the Sacroiliac Joint in Patients With Axial Spondyloarthritis

**DOI:** 10.3389/fimmu.2022.887470

**Published:** 2022-06-27

**Authors:** Yimeng Zhang, Zikang Guo, Lisong Wang, Ying Zhan, Jin Qu, Xinwei Lei

**Affiliations:** ^1^Department of Radiology, First Central Clinical College, Tianjin Medical University, Tianjin, China; ^2^Department of Radiology, Tianjin First Central Hospital, Tianjin Institute of Imaging Medicine, Tianjin, China; ^3^School of Basic Medical Sciences, Tianjin Medical University, Tianjin, China

**Keywords:** magnetic resonance imaging, axial spondyloarthritis, backfill, sacroiliac joint, sacroiliitis, fat metaplasia, spondyloarthritis research consortium of canada (SPARCC)

## Abstract

**Objectives:**

Fat metaplasia in an erosion cavity, also known as backfill, is an essential intermediary in new bone formation in axial spondyloarthritis (axSpA) patients; however, the predictors of backfill progression are unknown. This longitudinal study aimed to assess the predictors of backfill progression in axSpA patients on magnetic resonance imaging (MRI).

**Methods:**

Clinical and MRI data were collected at baseline and follow-up in 52 axSpA patients. Backfill progression was defined as the new or increased T1 hyperintensity within the SI joint space. Logistic regression analyses were performed to identify the predictors of the backfill progression.

**Results:**

A total of 19 patients had “backfill” at baseline and 30 patients exhibited the backfill progression after follow-up. The mean disease duration and SPARCC scores at baseline were significantly different between patients with and without backfill progression (*P*<0.001, *P*=0.003, respectively). Patients with backfill progression had a higher frequency of backfill at baseline, a higher SSS score of fat metaplasia, and a higher SSS score of backfill than those without (*P*=0.001, *P*<0.001, and *P*=0.002, respectively). A higher fat fraction value in the fat metaplasia area at the baseline was more frequent in patients with, than without, backfill progression (*P*=0.019). In the univariate logistic regression analyses, a higher SPARCC score for inflammation and a higher SSS score for fat metaplasia at baseline were associated with backfill progression.

**Conclusions:**

Severity of sacroiliitis and extensive fat metaplasia at baseline are predictors of the backfill progression in axSpA patients.

## Introduction

Axial spondyloarthritis (axSpA) is a chronic inflammatory rheumatic disease affecting the spine and the sacroiliac (SI) joints. The prominent feature of axSpA in the new bone formation is ankylosis in the SI joints and bridging syndesmophytes in the spine (1). This feature is associated with irreversible spinal immobility and functional limitation. Overall, new bone formation is a slow process in axSpA. Magnetic resonance imaging (MRI) is superior to radiography because it can demonstrate active inflammation and structural damages, even in patients in early axSpA with normal radiography findings. Fat metaplasia in an erosion cavity, also known as backfill, is a feature that can be detected on an MRI and is associated with new bone formation in the SI joints (1, 2). It has been defined as the presence of high signal intensity on T1-weighted images (T1WI) within the SI joint space and is hypothesized to represent reparative tissue refilling the eroded subchondral bone (2). Thus, identifying patients with a high likelihood of backfill progression is critical to adjusting the treatment strategy early in the disease process.

Inflammatory and chronic structural damages are objective signs of axSpA. Several studies have used the Spondyloarthritis Research Consortium of Canada (SPARCC) scoring system to evaluate the inflammatory status of SI joints (3). It is the most popular imaging standard of inflammation with high accuracy and reproducibility (4). Previous studies have established a correlation between SPARCC scores and clinical disease activity (5). Another MRI-based sacroiliitis activity index is the apparent diffusion coefficient (ADC) value from diffusion-weighted imaging (DWI), which detects the diffusion of water molecules and has been applied in the diagnosis and efficacy evaluation of sacroiliitis (6, 7). Fat metaplasia is frequently observed in the bone marrow after the resolution of inflammation in patients with axSpA. Previous studies have demonstrated that fat metaplasia is associated with an increased risk of spinal progression (8, 9). The mDixon-Quant sequence is one of the most accurate techniques to quantify bone marrow fat content by measuring the fat fraction (FF). FF is considered an alternative to invasive biopsy to study the distribution and quality of fat deposition (10, 11). Previous studies have investigated the usefulness of quantifying fat deposition by measuring fat fraction (FF) in patients with axSpA patients (12). In addition, a study suggests that FF estimation in the post-inflammatory area indicates the chronicity of axSpA and it is valuable in determining radiologic progression (13).

Only a few studies have explored predictors for backfill (14) and no studies have explored using quantitative MRI, which may provide important information for predicting new bone formation and prognosis in patients with axSpA. Therefore, this longitudinal study aimed to quantitatively assess the inflammatory and chronic structural damages and identify the predictors of backfill progression in axSpA patients.

## Materials and Methods

### Patients and Methods

For this study, patients with active sacroiliitis on a baseline MRI, clinical indications for tumor necrosis factor-alpha (TNF-α) inhibitor therapy, and no prior biologic therapy were selected. All patients were diagnosed with axSpA by an experienced rheumatologist according to the 2009 International Spondyloarthritis Assessment (ASAS) criteria (15). The clinical characteristics, including age, sex, symptom duration, human leukocyte antigen (HLA)‐B27 positivity, as well as the serum erythrocyte sediment rate (ESR) and C‐reactive protein (CRP) levels were obtained.

For patients who underwent TNF-α inhibitor treatment, the follow-up MRI scans were performed 6 and 12 months after baseline. Some patients were also followed up 3 months after treatment. Baseline and follow-up MRI scans were analyzed and evaluated by two radiologists independently. Once backfill occurs or progresses on MRI, the follow-up is stopped and classified as the progressive group. Backfill progression was defined as the new or increased T1 hyperintensity within the SI joint space (2). Those who did not present new backfill at 12 months or longer were classified as the non-progressive group. This study was approved by the Institutional Review Board of Tianjin First Central Hospital Medical Ethics Committee (2021N152KY). The patients provided their written informed consent to participate in this study.

### MRI Protocol

All patients were scanned using a 3.0-Tesla (T) MR scanner (Ingenia, Philips Healthcare, the Netherlands) with 32-channel, torso coils. Before DWI and mDixon-Quant, the routine sequences, including the T1WI mDixon-turbo spin-echo (TSE) oblique coronal plane (time of repetition (TR) 462 ms, echo time (TE) 16 ms, slice thickness 3 mm, slice gap 0 mm, matrix 276×210, field of view (FOV) 220 mm × 220 mm), T2WI mDixon-TSE oblique coronal plane (TR 2 300 ms, TE 90 ms, slice thickness 3 mm, slice gap 0 mm, matrix 276 × 219, FOV 220 mm × 220 mm), and T2WI mDixon-TSE axial section (TR 2 500 ms, TE 100 ms, slice thickness 3 mm, slice gap 0, matrix 276 × 215, FOV 220 mm × 220 mm). After scanning, T1WI and T2WI water images were automatically reconstructed and generated (i.e., fat suppression images). The DWI sequence was used for the SI joints in the axial plane with the following scan parameters: TR 3 015 ms, TE 62 ms, slice thickness 3 mm, matrix 86 × 86, FOV 220 mm × 220 mm, b value (0, 800mm/s^2^). Subsequently, the ADC map was created automatically. The mDixon-Quant sequence was used on the SI joints in the axial plane with the following scan parameters: TR 9.4 ms, TE 1.43 ms, ΔTE 1.3 ms, slice thickness 3 mm, FOV 220 mm × 220 mm, matrix 224 × 224, and using seven fat peak models for fat quantification. The system automatically reconstructed and generated in‐phase, out‐of‐phase, pure water, pure fat, R2* relaxation rate images, and FF map.

### Image Processing

DWI and mDixon-Quant images were processed using the Philips post-processing working station (Philips Intelli Space Portal). The regions of interest (ROIs) were delineated on the bone marrow edema (BME) and fat metaplasia areas according to conventional MRI, mDixon-Quant, and DWI (b=800s/mm^2^) on the automatically reconstructed FF and ADC maps. The criteria were as follows: BME lesions are located under the SI joint surface, with a high signal on the T2WI-mDixon water image and ADC map; the fat metaplasia lesions are located under the SI joint surface, with a clear margin and uniform high signal on T1WI. In the mDixon-Quant sequence, BME lesions showed a high signal on the water image, a low signal on the fat image; and the appearance of fat metaplasia lesions was opposite from the above.

The measurement method was as follows: a circular ROI (20-50 mm^2^) was marked at the center of the most extensive layer of the BME and fat metaplasia lesions on FF maps and ADC maps, avoiding the cortical bone and sclerosis area. If the patient had multiple lesions, each lesion’s ADC and FF values were measured separately and the average was considered the final ADC and FF values. If the patient had no fat metaplasia area at baseline, multiple ROIs were placed in the normal area than in the BME area and the average FF value was utilized.

### Standardized Assessment of MR Images and Scoring Methodology

MRI analysis focused on five types of lesions: BME (low signal intensity on T1WI and high signal intensity on T2WI located in the subchondral bone marrow); fat metaplasia (located under the SI joint surface with a clear margin and uniform high signal on T1WI); backfill (increased signal at a complete loss of the cortical bone clearly demarcated from the adjacent normal marrow by irregular dark signal); erosions (irregularly delineated joint space on T1-weighted sequences); and ankylosis (disappearance of the joint space on all sequences). MRI was considered positive for BME, fat metaplasia, backfill, or other structural abnormalities if the lesions were present on two consecutive slices or if two lesions were present on the same slice according to the ASAS definition (2).

All MRIs of active inflammation in the SI joints were evaluated for BME according to the SPARCC method (3). Then, the bilateral joint was divided into four quadrants: upper iliac, lower iliac, upper sacral, and lower sacral. The upper and lower halves of the SI joints were split on the coronal view. Each of these four quadrants was scored dichotomously. An additional score was given when both sides of the joint had a BME signal of ≥ 1 cm depth from the articular surface or presented an intense signal, respectively. The maximal score for a single coronal slice was 12 and the scoring system was repeated in six consecutive coronal slices, leading to a total score of 72.

All MRIs of chronic structural damages in the SI joints were evaluated for fat metaplasia, erosion, backfill, and ankylosis according to the SPARCC SSS method. The system was used to assess the structural lesions on T1WI (16). The presence/absence of lesions was scored in the quadrants (fat metaplasia and erosion) or halves (backfill and ankylosis). When assessing erosion and backfill, scaffolding was mutually exclusive for individual quadrants on individual images. The maximal score for a single coronal slice was 24 and the scoring system was repeated in five consecutive coronal slices, leading to a total score of 120. The scoring ranges were as follows: fat metaplasia (0-40), erosion (0-40), backfill (0-20), and ankylosis (0-20).

### Interobserver Agreement

In the event of a discrepancy between the two radiologists as to whether there was progression on the backfill, the final decision was made by a third senior expert. Baseline and follow-up MRI scans of the SI joints were scored independently by two radiologists blinded to all clinical data and MR time points. MRI SPARCC and SSS scores were assessed by two radiologists and the final score was the average of the two radiologists’ scores.

### Statistical Analysis

All data were statistically analyzed using SPSS 26.0 software. Continuous data were expressed as the mean ± standard deviation (SD) and categorical data as percentages. Normally distributed variables were compared using an independent t-test, while the non-normally distributed variables were compared using the Mann-Whitney U test. Categorical variables were compared between groups using the chi-squared test. Univariate logistic regression analysis was performed to identify the predictors associated with the progression of backfill.

Interobserver reliability of MRI SPARCC and SSS scores were assessed using the intraclass correlation coefficient (ICC). The correlation values were defined as follows: 0-0.2, poor; 0.3-0.4, fair; 0.5-0.6, moderate; 0.7-0.8, strong; >0.8, excellent agreement. *P*<0.05 indicated a statistically significant difference.

## Results

**Figure 1:**
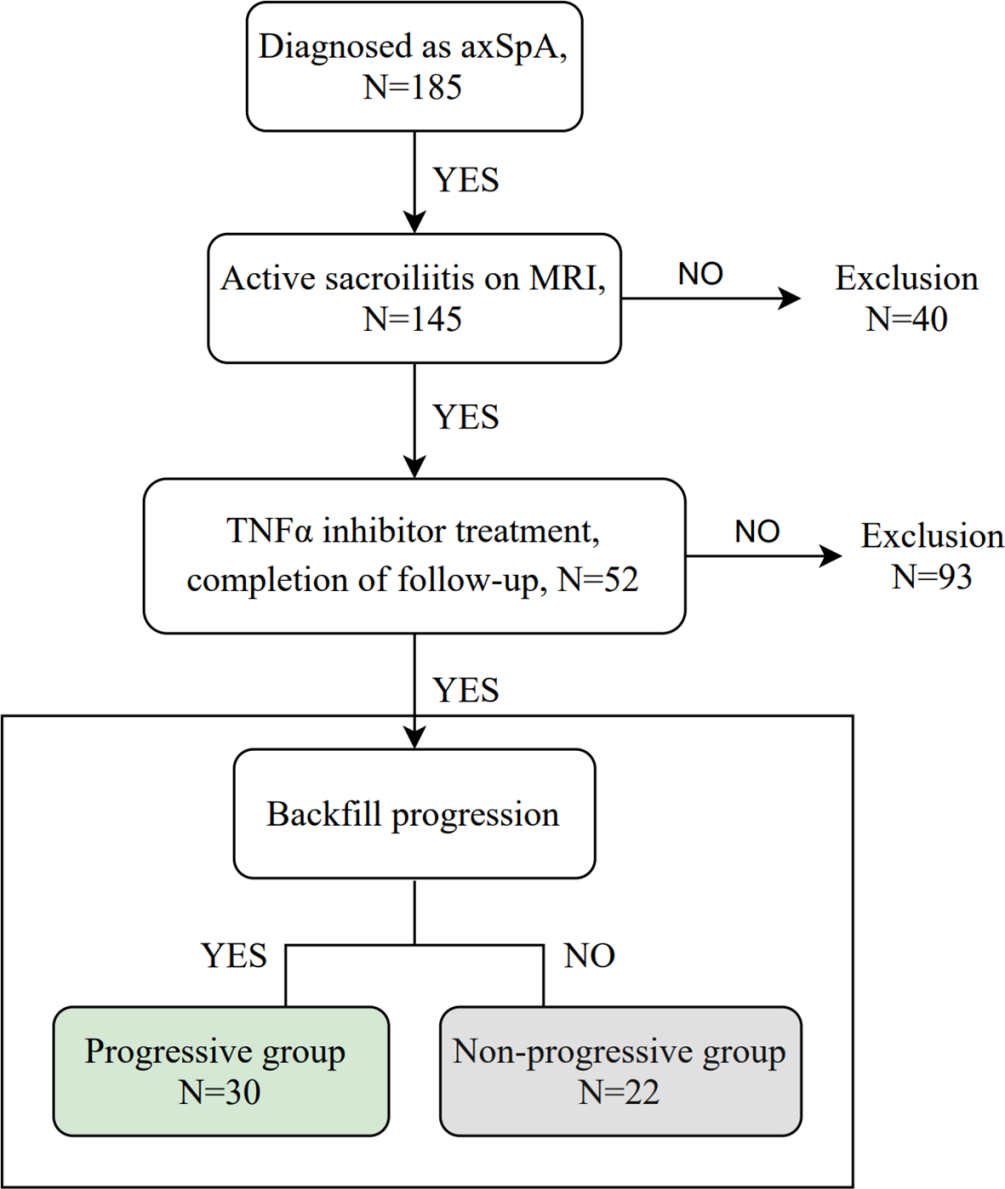
Flowchart of the study. axSpA, axial spondyloarthritis; MRI, magnetic resonance imaging; TNFα, tumor necrosis factor alpha.

**Figure 2:**
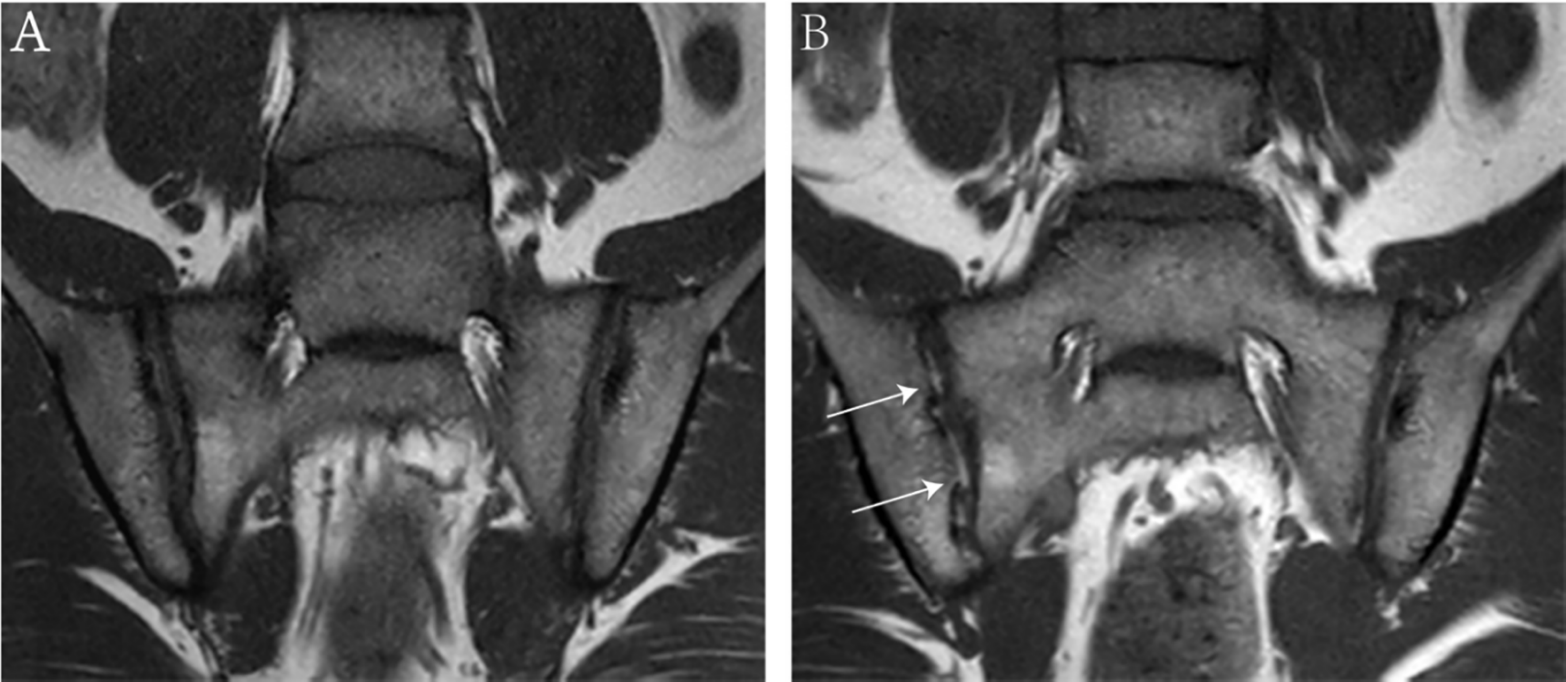
MRI findings of an 18-year-old male with HLA-B27 positive ankylosing spondylitis at baseline **(A)** and 6-month follow-up **(B)**. Coronal oblique T1 images show new ‘backfill’ at the right sacroiliac joints (white arrows) after 6-month follow-up.

**Figure 3:**
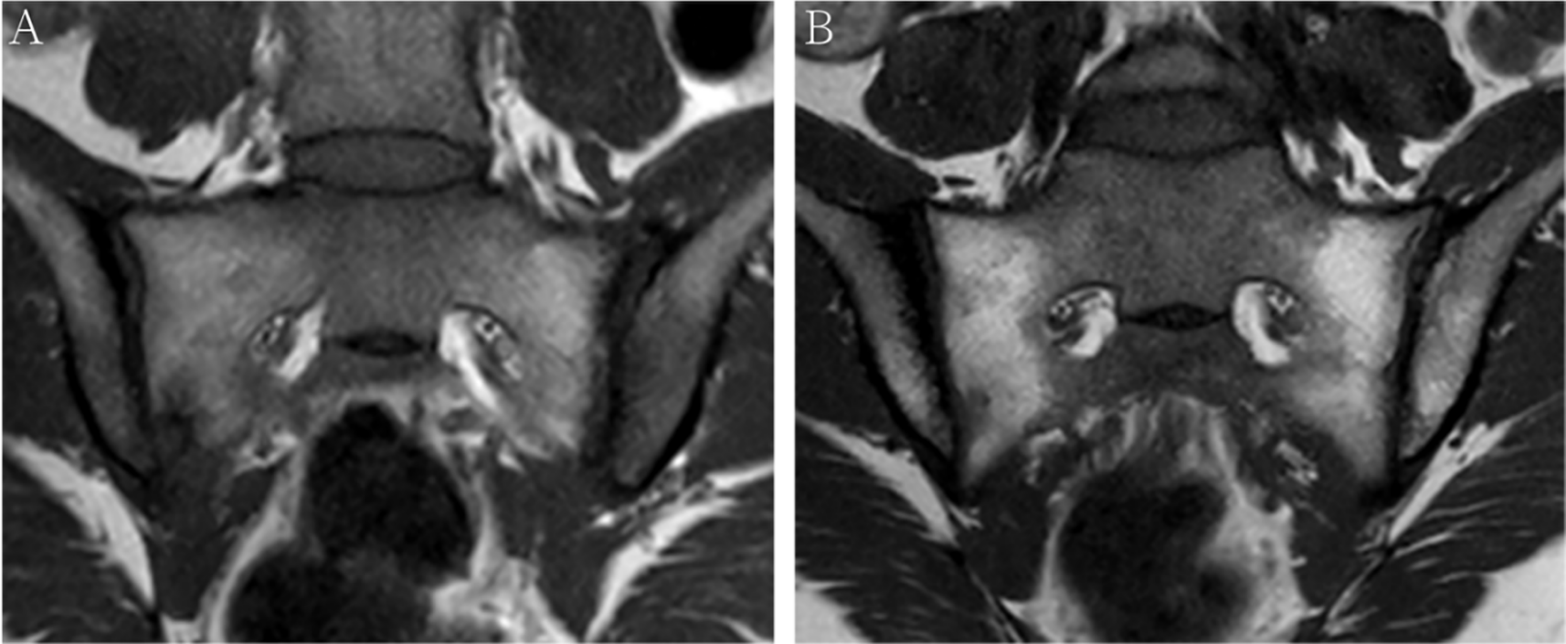
MRI findings of a 38-year-old female with HLA-B27 negative axial spondyloarthritis at baseline **(A)** and 12-month follow-up **(B)**. Coronal oblique T1 images show fat metaplasia at both sacroiliac joint surfaces, however, no backfill occurs after 12-month follow-up.

A total of 52 patients with axSpA who underwent baseline and follow-up MRI were evaluated (**Figure 1**). The mean age and disease duration were 25.94 ± 6.60 and 2.31 ± 1.55 years, respectively. 19/52 (36.5%) patients had “backfill” at baseline. After follow-up, new backfill progressed in 30/52 (57.7%) patients (**Figure 2**). One of them showed partial ankylosis during the 12-month follow-up. Fat metaplasia was observed in the periarticular bone marrow of 22 patients (**Figure 3**) but there was no backfill progression.

**Table 1** summarizes the baseline characteristics of the patients. The average follow-up periods were 5.9 and 17 months in the progressive and non-progressive groups. The mean age of the patients with backfill was slightly lower than those without backfill progression. However, our study group showed no statistical difference (*P*=0.274). The mean disease duration was higher in patients with backfill progression than in those without (*P*<0.001). No statistically significant differences were observed in CRP and ESR levels between the progressive and non-progressive groups.

**Table 1:** Comparisons of characteristics between axSpA patients with and without backfill progression at baseline.

Variables	Total axSpA patients (n = 52)	Progressive group (n = 30)	Non-progressive group (n = 22)	*P*-value
Age(years)	25.94 ± 6.60	25.03 ± 5.62	27.18 ± 7.69	0.274
Male	35 (67.3)	22 (73.3)	13 (59.1)	0.279
Disease duration(years)	2.31 ± 1.55	2.95 ± 1.47	1.43 ± 1.21	<0.001
HLA-B27 (+) ^*^	29 (74.4)	19 (82.6)	10 (62.5)	0.157
CRP (mg/L)	7.71 ± 14.06	8.45 ± 17.13	6.59 ± 7.62	0.578
ESR (mm/h)	16.98 ± 12.90	16.00 ± 14.31	18.47 ± 10.64	0.238
Baseline SPARCC	21.77 ± 15.83	27.47 ± 17.57	14.00 ± 8.53	0.003
Baseline SSS -fat metaplasia	13.29 ± 11.24	17.33 ± 10.85	7.77 ± 9.42	<0.001
Baseline SSS -erosion	4.85 ± 4.07	5.47 ± 4.76	4.00 ± 2.78	0.366
Presence of backfill	19 (36.5)	16 (53.3)	3 (13.6)	0.001
Baseline SSS -backfill	2.27 ± 4.15	3.67 ± 4.93	0.36 ± 1.29	0.002
Baseline ADC (×10^-3^ mm^2^/s) (inflammation)	1.13 ± 0.28	1.15 ± 0.23	1.11 ± 0.35	0.431
Baseline FF (%) (inflammation)	19.17 ± 12.37	19.87 ± 13.59	18.23 ± 10.71	0.839
Baseline FF (%) (fat metaplasia)	73.56 ± 13.28	77.45 ± 10.44	68.26 ± 15.05	0.019

axSpA, axial spondyloarthritis; HLA‐B27, human leukocyte antigen-B27; CRP, C‐reactive protein; ESR, serum erythrocyte sediment rate; SPARCC, Spondylarthritis Research Consortium of Canada; SSS, sacroiliac joint Structural scoring method; ADC, apparent diffusion coefficient; FF, fat fraction.

Values given as mean ± SD or n (%).

^*^Available in 39 patients.

**Figure 4:**
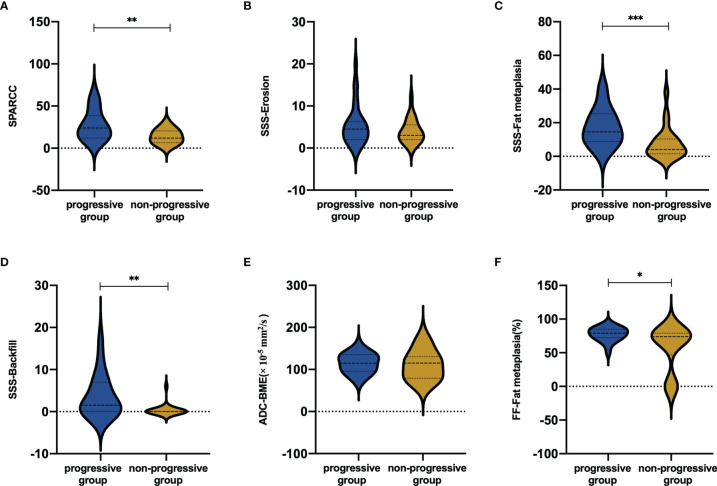
Mean scores in **(A)** BME, **(B)** erosion, **(C)** fat metaplasia and **(D)** backfill according to backfill progression or not. Mean values in **(E)** ADC and **(F)** FF values according to backfill progression or not. BME ranges in score from 0 to 72. Erosion and fat metaplasia range in score from 0 to 40. Backfill ranges in score from 0 to 20. *P < 0.05; **P < 0.01; ***P < 0.001.

The mean SPARCC score was 27.47 ± 17.57 (range: 7-66) and 14.00 ± 8.53 (2-30) in the progressive and non-progressive groups, respectively. The mean baseline SPARCC score was significantly different between patients with and without backfill progression (*P*=0.003). Moreover, the patients with backfill progression had a higher frequency of backfill at baseline, a higher SSS score of fat metaplasia, and a higher SSS score of backfill than those who did not develop new backfill (*P*=0.001, *P*<0.001, and *P*=0.002, respectively). No significant differences were detected in the baseline SSS score of erosion between the two groups (**Figure 4**).

The mean FF values of the progressive and non-progressive groups in the fat metaplasia area were 77.45 ± 10.44% and 68.26 ± 15.05%, respectively. A higher FF in the fat metaplasia area at baseline was more frequent in patients than in those without backfill progression (*P*=0.019). The mean ADC and FF values in the BME area of the progressive group were 114.6 ± 23.2 × 10^-5^ mm^2^/s and 19.9 ± 13.6%, respectively. Moreover, the mean ADC and FF values of the non-progressive group were 111.1 ± 34.9 × 10^-5^ mm^2^/s and 18.2 ± 10.7%, respectively. No significant differences were observed in the ADC and FF values in BME areas (**Figure 4**).

Univariate logistic regression analysis was performed to identify the factors associated with backfill progression of SI joints in patients with axSpA. The results showed that SPARCC score of inflammation [odds ratio (OR) 1.115, 95% confidence interval (CI): 1.031-1.205, *P*=0.006] and SSS score of fat metaplasia (OR 1.136, 95% CI: 1.042-1.240, *P*=0.004) differed significantly (**Table 2**).

**Table 2:** Univariate logistic regression analysis of the backfill progression.

Variables	OR (95% CI)	*P*-value
SPARCC score	1.115 (1.031-1.205)	0.006
Presence of backfill at baseline	0.243 (0.008-7.043)	0.222
SSS score of fat metaplasia	1.136 (1.042-1.240)	0.004
SSS score of backfill	1.904 (0.815-4.448)	0.070
FF value at fat metaplasia area	1.011 (0.962-1.063)	0.523

OR, odds ratio; CI, confidence interval; SPARCC, Spondylarthritis Research Consortium of Canada; SSS, sacroiliac joint Structural scoring method; FF, fat fraction.

MRI evaluation revealed a strong to excellent agreement between the two readers. An excellent correlation was achieved for the SPARCC and SSS assessment of inflammation, backfill, and fat metaplasia at baseline (ICC 0.83-0.91, 0.88-0.93, and 0.95-0.97, respectively). A strong to excellent correlation was established to identify erosion at baseline (ICC 0.78-0.90).

## Discussion

This study attempted to investigate the backfill progression in the SI joints and identify the potential risk factors of backfill in axSpA patients. About 57.7% of patients showed backfill progression over a 5.9-month follow-up interval. The results indicated that a high SPARCC score of inflammation and a high SSS score of fat metaplasia at baseline are predictors of the progression of backfill in axSpA patients. Furthermore, the mean disease duration, SPARCC score, SSS scores of fat metaplasia and backfill, frequency of backfill, and FF values in the fat metaplasia area at baseline were significantly associated with progressive backfill.

In 2019, the ASAS MRI working group introduced the new concept of backfill (2). Backfill was present in almost 66% of patients with spondyloarthritis (SpA) and has shown a high diagnostic value (17, 18). According to previous studies, fat metaplasia and backfill are early post-inflammatory changes and might reflect the early stage of bone remodeling (19, 20). The histology is not clear at this time. A previous study showed that the fat signal detected by spinal MRI of AS patients is related to a high content of adipocytes. Therefore, the disturbance of the homeostasis between osteoblasts and osteoclasts may be a crucial reason for new bone formation (21). A multivariate analysis of new bone formation in ankylosing spondylitis (AS) showed that fat metaplasia and backfill were significantly correlated with ankylosis (14). Typically, backfill indicates the initiation of new bone formation (1). However, no study has explored the correlation between fat metaplasia and backfill. Interestingly, we found that a high SSS score of fat metaplasia is significantly associated with the development of backfill. High FF values in fat metaplasia areas were frequent in patients with backfill progression, suggesting that fat metaplasia is a preceding stage of backfill. However, the histopathological analysis is not precise at this moment, which could be attributed to the effect of fat deposition on osteoblastogenesis in the bone marrow (22).

Subchondral bone destruction occurs after inflammation and reparative tissue fills the eroded area and progresses to ankylosis. A prospective study reported that baseline SI joints inflammation and backfill scores independently predict the 5-year progression of ankylosis (23). However, this study did not indicate the impact of the correlation between backfill and SPARCC scores. In the present study, a high SPARCC score predicted backfill progression in axSpA. This finding was consistent with a multivariable analysis that showed a correlation between backfill and SPARCC scores (24). This might be related to the expression of bone morphogenetic protein (BMP) genes. *BMP* gene is involved in chondrocyte differentiation and bone formation and the *BMP-2* gene promotes new bone formation. Another study of AS patients demonstrated that the expression level of the *BMP-2* gene was regulated by pro-inflammatory cytokines (such as TNF-α or interleukin (IL) -1β) and the higher the expression of the *BMP-2* gene, the faster the radiological progression of AS patients (25). Therefore, it is speculated that severe sacroiliitis, as detected on MRI, upregulates the level of the *BMP-2* gene, which further leads to the formation of new bone. Therefore, the degree of inflammation reflected by the SPARCC score is closely related to clinical treatment and prognostic stratification. Therefore, in patients with high SPARCC scores, targeted and aggressive therapy may be required to prevent further radiological progression. Both SPARCC score and ADC value are MRI sacroiliitis activity indices. The lack of correlation between ADC value in the BME area and the backfill progression may be because the SPARCC score reflects the extent, intensity, and depth of inflammation comprehensively, while ADC value reflects only the intensity of BME in a single area or it could be attributed to the absence of a uniform method for ROI delineation.

Delaying or preventing imaging progression is a major goal in the axSpA treatment process. Current studies have found that TNFi treatment can significantly improve inflammation and a study has shown that patients treated with TNFi have a lower rate of structural progression in the SI joints (26). All patients in this study were treated with TNF-α inhibitors. NSAIDs have been internationally recognized for the treatment of peripheral joint symptoms in axSpA; however, there is a lack of evidence for the drug’s therapeutic effect on axial skeleton disease and improving disease prognosis. Recently, IL-17 has become a hotspot research target in the field of axSpA treatment. IL-17 inhibitor secukinumab may have advantages in controlling and delaying the structural progression of axSpA (27), which needs to be verified by large-scale studies.

Nevertheless, the study has several limitations. Firstly, the sample size was small. Secondly, the patients in this cohort were followed up for only an average of 17 months. In the current study, the average follow-up period was 5.9 months in the progressive group and a previous study suggested that the change in backfill can be observed in 5 months (28). Long follow-up intervals would be influenced by several factors, such as self-discontinuation of medication by patients and recurrence of inflammation. Lastly, the ROI delineation was only a small, circular area, and defining ROI is methodologically challenging. Thus, the delineation of the full lesion is essential in future studies.

## Conclusion

The long disease duration, high SPARCC score, high SSS scores of fat metaplasia and backfill, high frequency of backfill at baseline, and high FF value in fat metaplasia area at baseline were frequent in patients with backfill progression. The severity of sacroiliitis and extensive fat metaplasia at baseline indicated a poor prognosis and an increased tendency of new bone formation in the SI joints.

## Data Availability Statement

The raw data supporting the conclusions of this article will be made available by the authors, without undue reservation.

## Ethics Statement

The studies involving human participants were reviewed and approved by Institutional Review Board of Tianjin First Central Hospital Medical Ethics Committee. The patients/participants provided their written informed consent to participate in this study.

## Author Contributions

YMZ, ZG, and XL contributed to the conception and design of the study. All authors performed the acquisition, analysis, and interpretation of data. YMZ wrote the manuscript. All authors reviewed the manuscript, and all authors approved the submitted version.

## Funding

Funded by Tianjin Key Medical Discipline (Specialty) Construction Project.

## Conflict of Interest

The authors declare that the research was conducted in the absence of any commercial or financial relationships that could be construed as a potential conflict of interest.

## Publisher’s Note

All claims expressed in this article are solely those of the authors and do not necessarily represent those of their affiliated organizations, or those of the publisher, the editors and the reviewers. Any product that may be evaluated in this article, or claim that may be made by its manufacturer, is not guaranteed or endorsed by the publisher.
